# The clinical features and related factors of PICC-related upper extremity asymptomatic venous thrombosis in cancer patients: A prospective study

**DOI:** 10.1097/MD.0000000000019409

**Published:** 2020-03-20

**Authors:** Guorong Wang, Yinfeng Li, Chunlin Wu, Lin Guo, Liqiong Hao, Hongyi Liao, Xue Xiao, Shanshan Liu, Lei Luo

**Affiliations:** Nursing Department, Sichuan Cancer Hospital & Institute, Sichuan Cancer Center, School of Medicine, University of Electronic Science and Technology of China, Chengdu, China.

**Keywords:** asymptomatic venous thrombosis, cancer patient, peripherally inserted central venous catheter, upper extremity thrombosis

## Abstract

Peripherally inserted central venous catheter (PICC) is the main venous access for cancer patients when they receive chemotherapy and nutritional support, but PICC-related venous thrombosis has become one of the most common and serious complications. It is very important to further explore the relationship among these features, so that prevent and treat the PICC-related thrombosis.

To investigate the clinical features and the related factors of PICC-related upper extremity asymptomatic venous thrombosis in cancer patients, and to provide theoretical basis for the prevention of venous thrombosis.

A total of 127 tumor patients with PICC catheterization were selected. Thrombus was detected by color Doppler ultrasound at different times: before catheterization and 24 hours after catheterization, and every week. The study was terminated at the time of thrombosis, and patients who did not develop thrombus were terminated after 6 weeks of follow-up. The clinical characteristics and influencing factors of asymptomatic thrombosis such as vessel diameter, blood flow velocity, thrombosis time, location, and the thrombosis stages were recorded.

The incidence of PICC-related upper limbs asymptomatic thrombosis was 48.82% (62/127), and the median time was 3 days. The incidence within 24-hour was 37.1% and within 1 week was 85.49%. A total of 81 venous thrombosis were found in 62 patients with asymptomatic thrombosis, there were 19 (23.5%) venous thrombosis in the deep veins while 62 (76.5%) in the superficial veins. Furthermore, thrombosis stages can be divided into 3 levels: stage I accounted for 51.85% (42/81), stage II accounted for 37.04% (30/81), and stage III accounted for 11.11% (9/81). The group trajectory analysis indicated the 3 changes of blood flow velocity during the follow-up period: downward trend, upward trend, and steady fluctuations. Survival analysis indicated that the cohort with downward trend have the high risk of thrombosis (67.90% vs 19.00% vs 45.10%). Cox proportional hazards model suggested that the patient's Eastern Cooperative Oncology Group score (hazard ratio [HR] 2.791, 95% confidence interval [CI] 0.08–0.76) and blood flow velocity (HR 0.250, 95% CI 2.01–3.87) was the risk of PICC-related asymptomatic thrombosis.

PICC catheterization can affect blood flow and asymptomatic thrombosis can occur at an early stage. Patient's upper limb activities should be guided to promoting blood circulation, thus effectively preventing thrombosis. Asymptomatic thrombosis can also be detected by color Doppler ultrasound system, within a recommended time of 1 week after catheterization.

**What is already known about the topic?**

PICC-related venous thrombosis occurs frequently in cancer patients and can induce a variety of complications. The 3 major causes of thrombosis have been identified. Lower extremity venous thrombosis occurs most often, but the clinical features, related factors, and relationships in PICC-related upper extremity asymptomatic venous thrombosis in cancer patients are unknown.

**Key points**

The incidence of asymptomatic venous thrombus after PICC differs, with the highest rate occurring during the early stages after catheterization.

The ECOG score was associated with the risk of asymptomatic thrombosis after PICC catheterization, and the higher the ECOG score the greater the risk of thrombosis.

The sudden change of blood flow at the early stage of catheterization puts the blood vessels in a “stress period," which is prone to thrombosis.

## 1 Introduction

The peripherally inserted central catheter (PICC) has been widely used clinically due to certain advantages such as its good safety record, high success rate, and long indwelling time, while it has also become the main venous access for cancer patients when they receive chemotherapy and nutritional support.^[1–3]^ However, a variety of factors, such as small peripheral venous diameters, slow blood flow, and general limb movement make PICCs vulnerable to thrombosis after placement in blood vessels. With the widespread use of PICC, PICC-related venous thrombosis has become one of the most common and serious complications with a frequency of 2% to 75%.^[3–7]^

According to the theory of Virchow's triad (circulatory stasis, vascular wall injury, hypercoagulable state) and Poiseuille's law, the placement of PICC can lead to intimal injury, which affects normal blood flow and blood flow velocity, thus increasing the risk of thrombosis. Researchers have explored risk factors for reducing thrombosis to reduce the damage to the intima during placement and indwelling, including catheter placement techniques; puncture sites; catheter tip position; catheter fixation and maintenance methods; patient personal factors; and drug treatment factors, with most studies having been focused on symptomatic thrombosis.^[1,3,4,8–11]^ Meanwhile, the ratio of the diameter of the catheter to the vascular caliber and the limb activity were also used to improve the blood flow velocity following placement.^[5,9,10]^ However, the present study indicates that other than the ratio of the catheter diameter and the blood vessel diameter, none of the other influencing factors involved the adaptation of the vessel (after PICC catheterization).^[9,10]^ Research is also limited regarding the changes in catheter diameter and blood velocity and the underlying factors of the thrombosis process, particularly the changes in blood flow velocity and the mechanisms affecting thrombosis.

Understanding the clinical features of PICC-related upper extremity asymptomatic venous thrombosis is therefore a prerequisite for the effective intervention of PICC-related thrombosis. This study aimed to observe the condition of the tumor patient during catheter placement and indwelling. Not only were the clinical features of asymptomatic thrombosis observed, such as the time, frequency, location, thrombus grade, vessel diameter, and blood flow velocity, but also an examination of the relationship among these features was necessary to determine a theoretical basis for the prevention and treatment of PICC-related thrombosis.

## 2 Patients and methods

### 2.1 Patients

The research subjects were 132 cancer patients who underwent chemotherapy at the Department of Radiotherapy and Oncology at the Sichuan Cancer Hospital between April 2014 and October 2014, and the cohorts were in line with the PICC requirements. The study was approved by the Ethics Committee of Sichuan Cancer Hospital and all patients signed informed consent. Inclusion criteria were as follows: patients undergoing PICC catheterization and catheter maintenance throughout the hospital; expected survival of more than 6 weeks; and patients with normal upper limb activities and muscle strength. Meanwhile, the exclusion criteria were: people with coagulopathy; patients who needed anticoagulation drugs due to underlying diseases; patients with a history of thrombosis; and patients who refused to join the study. The withdrawal criteria were: patients whose catheter maintenance was interrupted; unplanned extubation; patients with an aggravated condition or who automatically gave up treatment or died; and patients who dropped out of the study.

### 2.2 Materials and methods

#### 2.2.1 Equipment and device

(1) Portable ultrasonic apparatus: model (LOGIQ e), GE company GE Medical Systems (China) Co., Ltd.; probe frequency (6 ∼ 12 MHZ).
(2) PICC Catheter Line Kit: PICC with 3-way valve, model (7617405), size 4Fr (catheter diameter 1.4 mm) (Bard Groshong, Bard Medical Technology Co., Ltd, Murray Hill, New Jersey, USA). Open-ended PICC, model (384177), size 5Fr (catheter diameter 1.5 mm) (Bard Groshong). Modified Seldinger package: model (0668945), size 4Fr (Bard Groshong); model (384824), size 5Fr (Bard Groshong).

#### 2.2.2 Implementation

(1) Ethical review: the ethics committee of Sichuan Cancer Hospital reviewed and discussed this clinical research in strict accordance with the relevant regulations and guidelines and approved the study.
(2) Catheterization and maintenance: according to the standard process of PICC catheterization, all subjects’ PICCs were placed by qualified nurses in the central venous catheterization room. All catheters were performed using ultrasound guidance, and the vascular selection criterion was that the diameter of the catheter was ≤45% of the vascular diameter. The tip of the catheter was located at the lower third of the superior vena cava or at the junction of the superior vena cava and the right atrium.^[1,2,10]^ Catheter maintenance was performed in the PICC clinic by nurses who were trained and qualified.
(3) Ultrasound examination site: a trained nurse from the ultrasound examination department used Doppler ultrasound to examine the venous puncture site of the upper extremity, including the area 2 cm above the puncture site; 10 cm above the elbow fossa and the axilla; the subclavian vein and internal jugular vein.
(4) Ultrasound examination method: the patients were placed in a supine position and kept in a resting state, with their upper limbs abducted 90°, the palm upward, and the upper limb of the catheter fully exposed. The ultrasonic probe frequency was 6 ∼ 12 MHZ. First, the operator used two-dimensional ultrasound to display the transverse section along the vascular shape and measured the inner diameter of the blood vessel (D); ultrasound was then used to display the longitudinal section of the vascular shape. Blood flow was observed by color Doppler and the sampling volume was placed to be detected. At the center of the blood vessel, the angle between the ultrasound beam and the blood flow direction was ≤60°. The spectrum curve was recorded once the blood flow curve had shown a stable waveform and the average blood flow velocity (Vmean) was measured. If thrombosis was found, the patient was then transferred to the ultrasound department for diagnosis.
(5) Monitoring time: monitoring was performed at different times: before catheterization; 24-hours after catheterization; and at weekly PICC maintenance. If the ultrasound detected thrombosis, the study was terminated; if thrombosis did not occur, PICC was monitored until 42 days.
(6) Test indicators: blood vessel direction and branch, blood vessel inner diameter, and blood flow velocity. If thrombus formation was found, the time, location, and size were recorded, together with thrombosis stage and clinical manifestations.
(7) Data collection and recording: 2 nurses were selected for centralized training, including data filing and recording of thrombus morphology. Double verification was conducted during the recording phase to ensure the integrity and accuracy of the records.

#### 2.2.3 Thrombosis evaluation criteria

(1) Diagnostic criteria for asymptomatic thrombosis^[6,9]^: the color Doppler ultrasonography revealed that the upper extremity lumen was found to be full, with a low echo or echogenic mass in the lumen. However, there was no upper limb swelling, tenderness of the catheterization site or adjacent site, elevation of skin temperature, skin cyanosis, limb sensation and dysfunction, or shoulder discomfort. The venous lumen probe did not collapse and no obvious blood flow signal was observed.
(2) Venous thrombosis stages^[12]^: stage I: small echoes of small clumps and/or small wall-shaped masses (i.e., thrombi) were visible in the venous lumen but were mainly isolated. Color Doppler blood flow imaging (CDFI) showed sound venous blood flow and a narrow vascular cross-section ranging from 1% to 30%. Stage II: thrombus formation was observed in the venous lumen and/or around the catheter with multiple sites. CDFI showed sound venous blood flow and cross-sectional narrowing of the vessel reached 31% to 50%. Stage III: multiple thromboses were observed in the venous lumen and around the catheter with the fusion type being predominant. CDFI showed eddy currents in the blood flow, which was effectively unobstructed, and the cross-sectional area of the blood vessel was narrowed by 51% to 70%. Stage IV: a large venous thrombosis was observed in the venous lumen and the large-area lumen was filled with thrombi. CDFI only showed part of the blood flow signal passing through the narrow passage. The cross-sectional narrowing of the blood vessel ranged from 71% to 99%. Stage V: vein occlusion, the vessel lumen was filled with thrombosis, and CDFI showed no blood flow signal.

### 2.3 Statistical method

Statistical description: the count data was described by percentage and the measurement data was described by mean and standard deviation. Statistical analysis: the group trajectory analysis and survival analysis of blood flow velocity were performed by R language. The risk of thrombosis in each category was compared by dividing the population into different categories according to the average blood flow velocity trend. The survival analysis was performed with or without thrombus. The Cox proportional hazard model was used to analyze the influencing factors of thrombosis.

## 3 Results

### 3.1 General information

Over the study period, 132 PICCs were inserted, 5 of which were abandoned as a result of disease exacerbation, so the effective sample size was 127. All patients had PICCs placed once and inserted successfully, with the position of the catheter tip being in line with requirements and normal function indicated; all PICCs were fixed with a sutureless catheter dressing. The average age of the participants was 52.69 ± 12.00 years old and males accounted for 53.5%; about one-third of patients received a tumor resection before chemotherapy. Demographic data, disease, and venous access-related data are shown in Table 1.

**Table 1:**
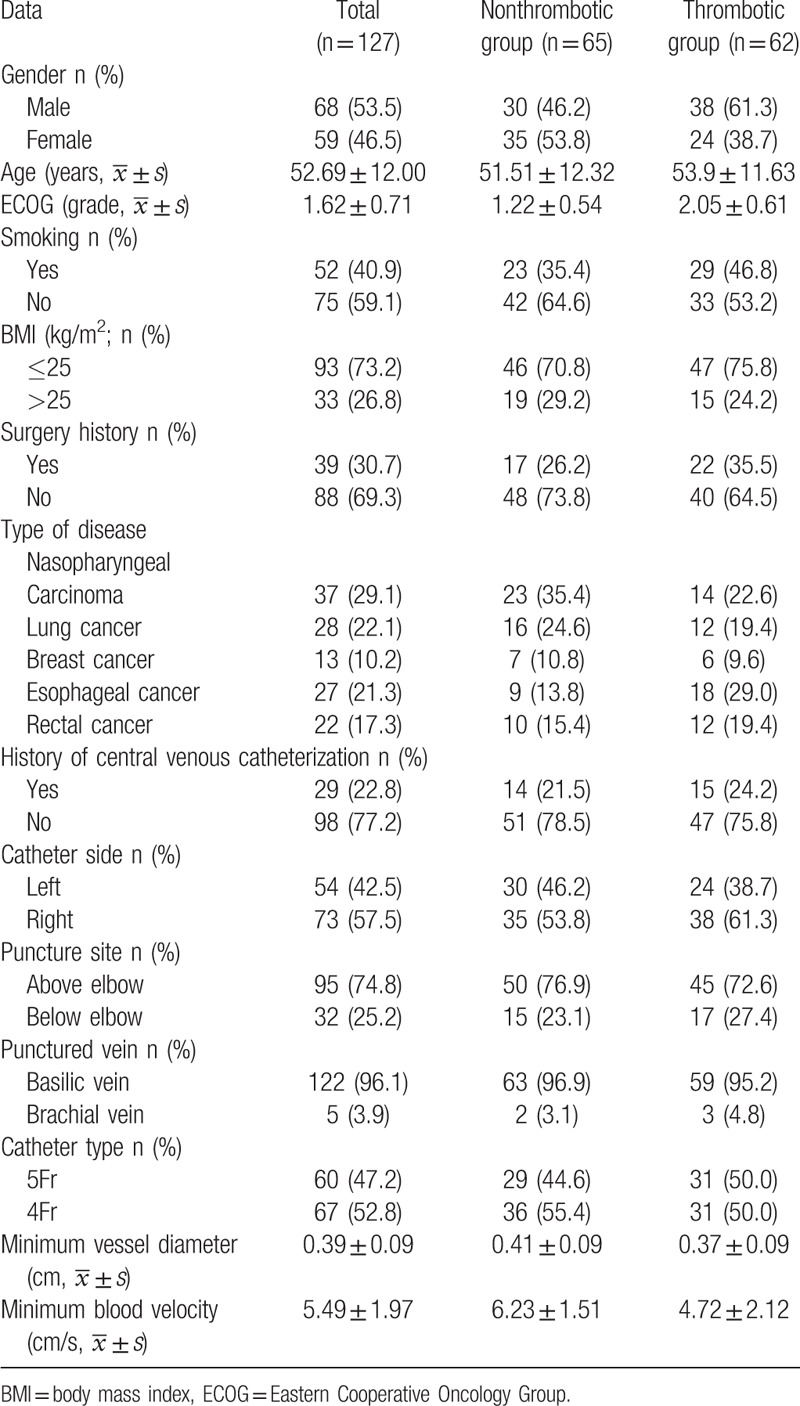
Demographic, disease, and venous access data of the study population.

### 3.2 Clinical features of asymptomatic thrombus

Among the 127 patients who were followed up for 42 days, 81 asymptomatic thromboses were detected, which were mainly distributed in the superficial perivascular vein (basilica vein) (76.54%), and a proportion in the cephalic vein (22.22%). In this study, only 1 case involved 3 sites at the same time, and neither the jugular vein nor the cephalic vein was involved. Stage I and II thrombosis dominated, accounting for 88.89% (Table 2). The median time for asymptomatic thrombosis was 3 days, the earliest was 24 hours after catheterization, and reached more than one-third (23, 37.10%); the latest was after33 days. Among them, 85.49% (53/62) occurred within 1 week of catheterization, and the most frequently involved site was the basilica vein within 10 cm of the puncture site (Table 2).

**Table 2:**
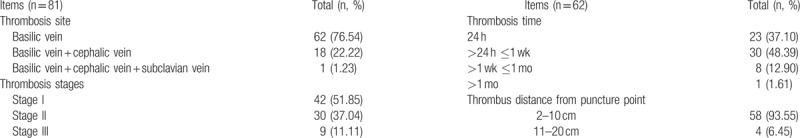
Clinical features of asymptomatic thrombus.

### 3.3 Effect of blood flow velocity on asymptomatic thrombosis

**Figure 1:**
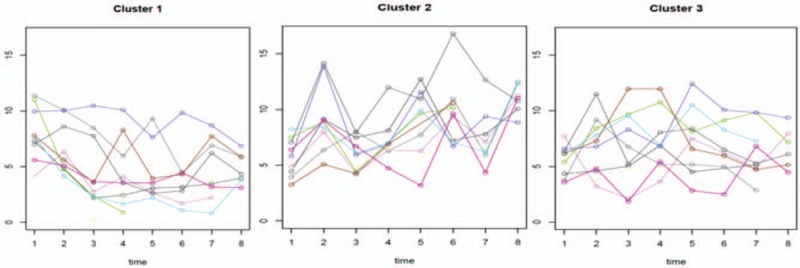
After catheterization, we performed group analysis on the fluctuation of blood flow velocity, and divided the participants into 3 groups according to the results of group analysis. Cluster 1 was the group with decreased blood flow velocity, cluster 2 was the group with increased blood flow velocity, and cluster 3 was the group with steady blood flow velocity.

**Figure 2:**
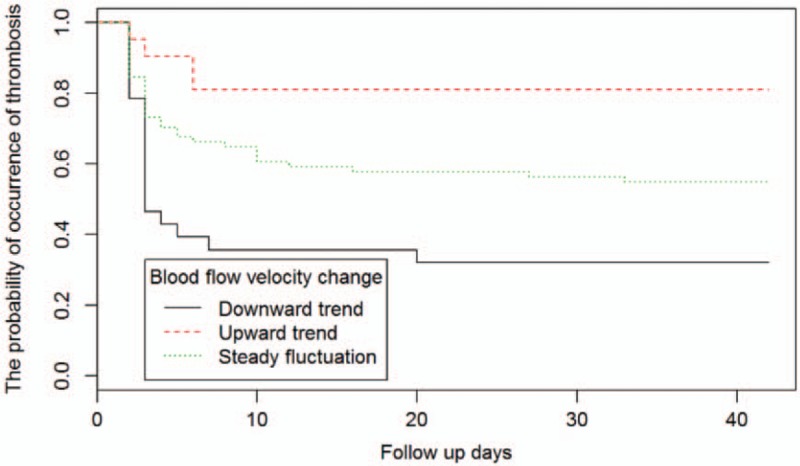
According to group analysis, we performed survival analysis on the probability of occurrence of thrombosis. The abscissa indicated time and the ordinate indicated the possibility of thrombosis. The smaller the number, the greater the possibility of thrombosis, and 1 indicates no occurrence. It can be seen that the group with upward trend was much less likely to have thrombosis than the group with decreased blood flow velocity. In addition, the 3 groups were most likely to have thrombus within 1 week after catheterization (approximately 7 days).

According to the changes in blood flow at different detection points and different times, the lowest blood flow velocity of each test was taken to analyze the group trajectory (7 cases were excluded due to missing single data). According to the group trajectory analysis, blood flow velocity changes indicated 3 states during the follow-up period: downward trend (cluster 1), upward trend (cluster 2), and steady fluctuations (cluster 3). Combined with survival analysis of thrombosis in different types of blood flow velocity, there was a difference in the incidence of thrombosis in different blood flow trend groups, with the downward trend cohort having the highest risk of thrombosis (Table 3, Figs. 1 and 2).

**Table 3:**
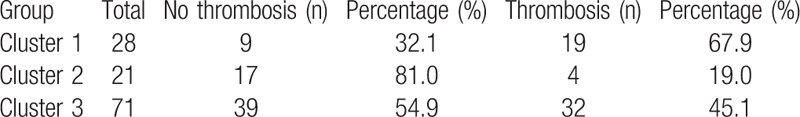
Thrombosis rate in different groups (n = 120).

### 3.4 Analyzing the influencing factors of asymptomatic thrombosis

Univariate and multivariate factors for PICC-related thrombosis were studied by performing the Cox proportional hazards model. The lowest inner diameter of the blood vessel was used, and the blood flow velocity was analyzed by the results of the group trajectory analysis. The results of the Cox proportional hazards model suggested that there was a relationship between the Eastern Cooperative Oncology Group (ECOG), declined venous flow velocity, and PICC-related upper extremity asymptomatic venous thrombosis (*P* < .05). There was no statistical significance in the influence of gender, age, body mass index, history of surgery, history of central venous catheterization, catheterization limb, puncture site, puncture vein, catheter type, or vessel diameter (Tables 4 and 5).

**Table 4:**
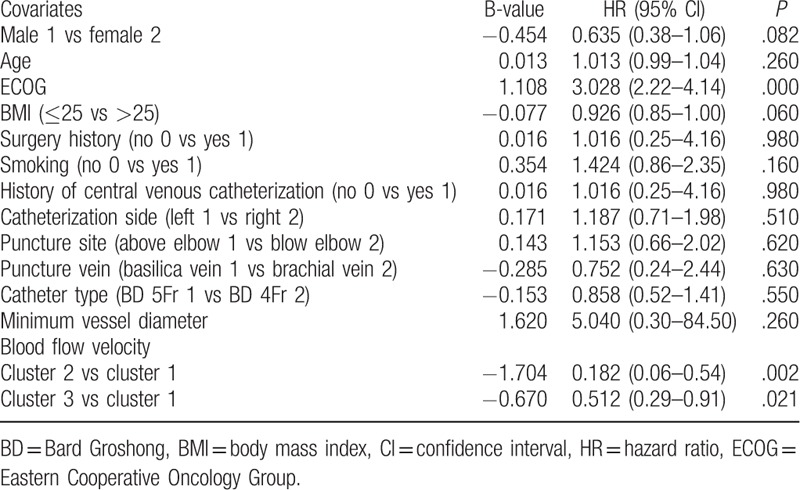
Analysis of univariate factors affecting peripherally inserted central venous catheter asymptomatic venous thrombosis (n = 127).

**Table 5:**
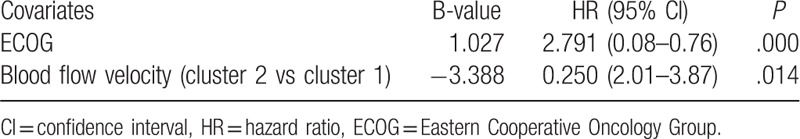
Analysis of multivariate factors affecting asymptomatic thrombosis.

## 4 Discussion

### 4.1 Prevention of PICC thrombosis in cancer patients should begin early

Asymptomatic thrombosis mainly occurs in superficial veins and may be accompanied by deep vein thrombosis, while other characteristics are a high incidence rate and early occurrence time.^[7–9]^ The research indicated that the rate of asymptomatic thrombosis during the 6-week follow-up period was 48.82% (62/127). Menéndez et al’ study^[9]^ and other reports advise that the rate of asymptomatic thrombosis in children was 27.5%, but all of them were much higher than the symptomatic thrombosis rate of 23.54%.^[4,7]^ The study took place at an earlier stage than the other studies, showing that the incidence of asymptomatic thrombosis within 24 hours reached 37.10%. The continuous detection process indicates a significant reduction in the incidence of asymptomatic thrombosis over time, with the peak period being within 1 week (85.49%, median time was 3 days); while the incidence rate was only 12.90% after 1 week, and only 1.61% after 1 month (Table 2, Fig. 2). Present studies have shown that the median time of asymptomatic thrombosis in PICC is 1 to 2 weeks earlier than symptomatic thrombosis.^[7,9,11]^ Clinically-observed studies by Luo et al^[7]^ found that 50% of symptomatic thrombosis occurred 2 to 3 weeks after catheterization; while only 32.14% occurred within 1 week, and asymptomatic thrombosis reached 85.49%. The results indicated that there was a “stress period" of vascular adaptation for 1 week following catheterization; asymptomatic thrombus is the adaptation process and outcome of PICC catheterization; some asymptomatic thrombosis can be adaptive to the body without symptomatic thrombosis; symptomatic thrombosis can occur when this is maladaptive.^[7,9]^ Chopra et al^[8]^ believes that PICC-related deep vein thrombosis is a concomitant or progressive outcome of superficial venous thrombosis, while the risk of PICC-associated deep vein thrombosis can be reduced by preventing superficial venous thrombosis. This study suggested that 51.85% of stage I and 88.89% of stage I and II asymptomatic thrombosis is dominated by microthrombosis. Therefore, improving the adaptability of blood vessels to the catheter and enhancing the body's resistance to the risk of thrombosis is beneficial to the body's removal of tiny thrombi and the reduction of symptomatic thrombosis. Therefore, the most significant period to prevent thrombosis is within 1 week following PICC catheterization, so it should be carried out immediately after catheterization to improve the preventive effect.

### 4.2 Effect of venous flow velocity on asymptomatic thrombosis

Although this study strictly followed a reasonable ratio of catheter diameter to vessel diameter, ensuring that the vessel had sufficient space to meet the blood flow velocity requirements; the lowest blood flow velocity in the thrombotic group was lower than in the nonthrombotic group (Table 1). There are differences in blood flow velocity changes between different individuals. According to the group trajectory analysis of average blood flow velocity at 7 time points during the follow-up period, the changes to blood flow velocity following catheterization showed a trend of “downward, upward, and smooth": 23.33% of patients showed a downward trend; 59.17% of patients remained stable; 17.5% of patients had a slow rise in blood flow rate (Table 3, Fig. 1). Survival analysis indicated that the downward group had the highest risk of thrombosis while the upward group had the lowest risk (Table 3, Figs. 1 and 2). Wilson et al's study^[13]^ and other studies claim that catheterization occupies a certain space in the venous lumen, and as the catheter directly hinders the blood flow, changes to blood flow state and blood flow persistence are the basis of thrombosis, rather than vascular injury.^[14,15]^ According to the study, after ensuring a reasonable ratio of the diameter, the blood flow velocity of most patients before and after catheterization maintained a steady or even rising trend, indicating that the body itself has a certain ability to adapt and adjust. However, the results of this study also indicated there was a decreased blood flow velocity trend in 23.33% of patients, while the thrombosis rate was higher in the downward group and the smooth group, with only the upward trend group evidencing a protective factor for thrombosis. Therefore, measures to promote blood flow should be considered in the prevention of PICC thrombosis. In high-risk populations, or when conditions permit, ultrasound dynamics can be used to assess the blood flow velocity before and during indwelling, so that the blood flow velocity maintains an upward trend, thus reducing thrombosis.

### 4.3 Effect of patient activity on asymptomatic thrombosis

According to this study, a higher ECOG score indicates poorer activity status and a higher risk of asymptomatic thrombosis. Therefore, attention should be given to assessing the patient's activity status and ability before catheterization. In addition, PICCs in cancer patients with poor activity should be carefully placed, while targeted limbs should be promoted to perform the purpose activity so that blood circulation is promoted after catheterization and during catheter indwelling.^[2,14]^ The ECOG score reflects the patient's activity status and general health status, as well as being a recognized indicator of patient activity and tolerance to treatment.^[16]^ Rui et al^[17]^ claims that the risk of asymptomatic thrombosis in patients with reduced activity is 1.6 times higher than those with normal activity. The main reason for this is that poor physical status and reduced activity can lead to slow blood flow velocity and even blood stasis, which leads to thrombosis. However, clinical focus has largely been directed toward the effect of active status on spontaneous deep venous thrombosis, while less attention has been paid to PICC-related upper extremity thrombosis. In terms of cancer patients, there are significant ECOG changes due to the side effects of treatment and changes in the patient's condition. When there is a longer PICC indwelling time, it is easy for clinical staff to ignore the risk of thrombosis during PICC indwelling. Although this study also suggests that the indwelling time is more than 1 month, the incidence of asymptomatic thrombosis is extremely low. However, in view of the important influence of activity status on thrombosis, special attention should be paid to the dynamic assessment of patients with PICC, while patients with decreased activity should be given comprehensive thrombosis protection.

Therefore, an evaluation of activity ability should be taken into account during catheterization and indwelling. Limb movement should be enhanced after catheterization and during indwelling for the purpose of promoting blood circulation. This principle has been incorporated into the Infusion Nursing Society, where a complementary method of making fists has been developed.^[2,14]^ Due to the important influence of activity status on thrombosis, clinical practice not only requires preventive fist movement but should also pay special attention to the dynamic assessment of patients with PICC, and thrombosis prevention should be enhanced when the patient's activity declines. It is recommended that the quantitative relationship between activity volume and the improvement of blood flow velocity should be studied further in order to achieve precise guidance.

In conclusion, asymptomatic thrombosis is a common phenomenon after PICC catheterization, while activity state and declined blood flow velocity are significant factors in PICC-related upper-extremity asymptomatic venous thrombosis. Therefore, the patient's activity status and venous flow rate should be adequately screened and evaluated before and after catheterization. Following PICC catheterization, medical staff should scientifically guide the patient's upper limb activities, as well as providing effective measures to promote blood circulation, thus effectively preventing thrombosis. Under the right conditions, asymptomatic thrombus may also be detected at an early stage by use of the color Doppler ultrasound system, with the preferable detection time being 24 hours to 1 week after catheterization.

## 5 Limitations and future implications

This study was a single-center study with a small sample size and a short follow-up time. Future research should not only enable a multicenter and full follow-up model but will also study the predictive ability of blood flow velocity to thrombosis and the clinical outcome of asymptomatic thrombosis.

## Acknowledgments

The authors would like to thank the staff in the department of central venous catheter room, who helped in collecting data. Many thanks to C.W. for his advice on data statistics and the handling of SPSS. We also would like to thank the Department of Ultrasound for its help in imaging. Finally, we would like to thank the Scientific Research of Sichuan Province Health Department for the financial support.

## Author contributions

**Conceptualization:** Guorong Wang, Yinfeng Li.

**Data curation:** Guorong Wang, Yinfeng Li, Chunlin Wu, Hongyi Liao, Xue Xiao.

**Formal analysis:** Liqiong Hao.

**Methodology:** Chunlin Wu, Shanshan Liu.

**Project administration:** Lin Guo, Lei Luo.

**Resources:** Liqiong Hao, Hongyi Liao, Xue Xiao, Shanshan Liu, Lei Luo.

**Software:** Lin Guo.

**Supervision:** Lei Luo.

**Validation:** Lei Luo.

**Writing – original draft:** Guorong Wang, Yinfeng Li.

**Writing – review & editing:** Guorong Wang, Yinfeng Li.
